# Acute Parkinsonism and Cerebral Salt-wasting-related Hyponatremia in Scrub Typhus

**DOI:** 10.7759/cureus.6706

**Published:** 2020-01-20

**Authors:** Sunitha Soundararajan, Stalin Viswanathan, Dheeraj Jain, Vijayalatchumy Krishnamurthy, Murugesan S Gayathri

**Affiliations:** 1 General Medicine, Indira Gandhi Medical College and Research Institute, Pondicherry, IND; 2 General Medicine, Jawaharlal Institute of Postgraduate Medical Education and Research (JIPMER), Pondicherry, IND; 3 General Medicine, Indira Gandhi Medical College & Research Institute, Pondicherry, IND; 4 Internal Medicine, Indira Gandhi Medical College & Research Institute, Pondicherry, IND; 5 Radiology, Indira Gandhi Medical College & Research Institute, Pondicherry, IND

**Keywords:** scrub typhus, parkinsonism, cerebral salt wasting syndrome

## Abstract

Scrub typhus is a multisystem disease, and the respiratory system is commonly involved. Scrub typhus-related parkinsonism has been reported in three patients previously, and none of them underwent a lumbar puncture. Cerebral salt wasting is generally observed in vascular emergencies of the brain. We report a patient with acute parkinsonism and cerebral salt wasting syndrome, a combination of which has not been previously reported in scrub typhus. A 50-year-old farmer presented with an acute febrile illness of two weeks’ duration and a one-day history of parkinsonism symptoms. His workup revealed hypoosmolar hyponatremia, increased urinary sodium excretion, hepatorenal and hematological dysfunction, and normal findings on cerebrospinal fluid examination. Clinical and biochemical improvement was noticed with the institution of doxycycline. Empirical doxycycline may be needed in patients with acute parkinsonism following an acute febrile illness in areas endemic for scrub typhus. Better biochemical facilities in resource-poor tropical countries would help in evaluating the etiology of hyponatremia due to infectious causes.

## Introduction

Scrub typhus is a zoonotic infection that causes a nonspecific febrile illness with multisystem manifestations, most commonly involving the respiratory system. Various neurological manifestations of scrub typhus have been reported in case reports and case series [1,2]. Parkinsonism has been reported in three instances previously, none of whom underwent a lumbar puncture [3-5]. Cerebral salt wasting (CSW) is a disorder generally described in patients with vascular emergencies of the brain. We report a middle-aged farmer with acute parkinsonism following an acute febrile illness and was detected to have hyponatremia due to CSW.

## Case presentation

A 50-year-old farmer from the neighboring district visited the emergency department with 15 days’ history of fever, productive cough and breathlessness, and acute onset of slurred speech, tremors, insomnia, and disorientation since the previous day. His past history was insignificant for chronic diseases. He was a chronic smoker (30 pack-years) and alcohol user, but had stopped both of them since the onset of fever. He had traveled to a nearby village for a sugarcane harvest before the onset of fever. He had not taken any drugs prior to his hospital visit, except for acetaminophen. On examination, he was febrile (38.5°C), with pulse 104/min, blood pressure 80/60 mmHg, respiratory rate 24/min, and had small bilateral axillary lymph nodes, bilateral rhonchi on respiratory examination, and mild hepatomegaly. He was disoriented to time, with bilateral resting tremors of his hands, mask-like facies, and slow and slurred speech which was hypophonic. Observing his gait 24 hours after volume replenishment showed that he walked slowly with short steps, difficulty in turning, and reduced arm swing. There were no falls or other focal neurological deficits. 

Intravenous fluids (normal saline) were administered, which led to a marginal improvement in his blood pressure to 90 mm systolic; he required a blood transfusion of nearly 5 L within the first 36 hours. He refused a central venous pressure line. Electrocardiogram and bedside echocardiography were normal. Chest radiography was reported to be normal. Investigations in the casualty revealed prerenal azotemia, hyponatremia (hypovolemic hypoosmolar-255.58 mosm/L), mild thrombocytopenia, and neutrophilia. Plain computed tomography of the head showed a parietal calcified granuloma. Pending cultures and serological tests (dengue IgM, scrub IgM, and Widal, human immunodeficiency virus, and hepatitis B surface antigen), he was initiated on ceftriaxone and doxycycline considering the high local prevalence of scrub typhus. His investigations revealed mild elevation of transaminases, normalization of renal function, persisting hyponatremia with increased urinary sodium (performed 48 hours later), normal uric acid, thyroid-stimulating hormone, and negative serological tests except for scrub IgM. He could not afford cortisol testing, which was not available in our institution. Consent for lumbar puncture was given by his wife only 24 hours after admission, and cerebrospinal fluid (CSF) studies were normal (Table 1).

**Table 1:** Lab investigations of the patient AST, aspartate aminotransferase; ALT, alanine transaminase; TSH, thyroid-stimulating hormone; ADA, adenosine deaminase; CBNAAT, cartridge-based nucleic acid amplification test.

Test		Day 1	Day 2	Day 5	Day 7
Hemoglobin (14-16 g/dL)		14.2	13.1		
Total leukocyte counts (4-10 x10^9^/L)		6,000	7,600		
Neutrophilia (%)		73	84		
Platelets (150-450 x10^9^/L)		141	176		
AST (12-38 U/L)			61	42	
ALT (7-40 U/L)			71	24	
Albumin (4-5 g/dL)			2.9	2.3	
Sodium (135-145 mmol/L)		120	128	128	130
Potassium (3.5-4.5 mmol/L)		3.9	3.3	3.3	3.5
Urea (7-20 mg/dL)		69	27	14	
Creatinine (0.6-1.2 mg/dL)		1.4	0.9	0.7	
Creatine kinase (50-290 U/L)		96	86		
TSH (0.5-5 µIU/mL)		2.1			
Amylase (20-120 U/L)		119			
Urinalysis		Normal			
Urine sodium (<40 mmol/L)		89			
Cerebrospinal fluid examination on day 2					
Cells	5	Differential counts	All lymphocytes		
Protein	21.8 mg/dL	Glucose	85 mg/dL	Blood glucose	104 mg/dL
ADA	3.4 U/L	CBNAAT	Negative		
Gram stain	Negative	Culture	Sterile		

He became afebrile within 48 hours. Three liters of normal saline was given daily until the third day when he began taking his usual diet and his serum sodium stabilized at ~130 mmol/L. He became fully oriented and began smiling on the fifth day. Tremors improved on day 7 without the use of beta-blockers. On the eighth day, his gait became normal with his usual arm swing, and his hypophonia and slowness of speech improved. He was discharged on day 10 to complete a 14-day course of doxycycline given his neurological involvement. On his follow-up one week later, his neurological symptoms had recovered completely, and serum sodium was 136 mmol/L.

## Discussion

The involvement of the central nervous system occurs hematogenously from the peripheral cells of the monocyte-phagocyte system that the rickettsiae parasitize [1]. Leptomeningeal infiltration, CSF invasion, perivasculitis, parenchymal inflammation and infarction, typhus nodules, and demyelination lead to clinical features in scrub typhus [2]. Meningitis is the most common neurological manifestation. Others include cranial neuropathies, plexopathies, peripheral neuropathies, Guillain-Barre syndrome, transverse myelitis, acute disseminated encephalomyelitis, cerebral infarction, subdural hematoma, subarachnoid hemorrhage (SAH), cerebral venous thrombosis, seizures, coma, cerebellitis, neuroleptic malignant syndrome, and psychiatric manifestations [1,2] Parkinsonism has been described in three cases, who were all from rural areas [3-5]. Two were elderly males, while the third patient was a middle-aged man like ours. One man had an infarction of the basal ganglia and cerebellum, and mild elevations of transaminases and thrombocytopenia [4]. The first reported patient also had myoclonus and had been treated with amantadine and clonazepam [3]. All of them improved quickly, over days to weeks. A mask-like facies also made us consider hypothyroidism and depression which were ruled out.

Scrub typhus with CSW has been reported only once in the medical literature, but the patient, an elderly female, also had SAH which in itself is a cause of CSW [6]. Hyponatremia as in our case, with fever and neurological involvement, could be caused by either CSW or syndrome of inappropriate antidiuretic hormone secretion (SIADH) since there was no history of fluid loss in the form of diarrhea, vomiting, or polyuria. However, the presence of hypotension at admission (volume depletion is the most important differentiating factor between CSW and SIADH), hypovolemic hypoosmolar hyponatremia, mild hemoconcentration, and prerenal azotemia, associated with increased urinary sodium (performed 48 hours after admission with ongoing sodium correction) and low normal uric acid in the setting of neurological symptoms and signs, favored a diagnosis of CSW [7]. Hyponatremia could have also contributed to his disorientation and gait abnormalities. Other causes of hyponatremia including hypothyroidism were ruled out. Cortisol and urine osmolality could not be done due to unavailability in our institution. Lumbar puncture ruled out other local infections and SAH. Hypoalbuminemia as in our patient has been described to be associated with complications and a longer hospital stay due to scrub typhus infection [8].

## Conclusions

In areas with high prevalence, scrub typhus must be remembered as a cause of acute febrile illness with neurological signs. A combination of acute parkinsonism and CSW has hitherto remained unreported in scrub typhus. Though serology may be positive, neuroimaging and CSF studies are still required to rule out other secondary causes, the latter test not having been done in any of the three previously reported patients. Biochemical facilities for urine samples in resource-poor tropical settings such as ours would go a long way in detecting infectious CSW, a diagnosis commonly confused with SIADH.
